# Effects of social support in an academic context on low-grade inflammation in high school students

**DOI:** 10.1007/s10865-021-00241-x

**Published:** 2021-08-06

**Authors:** Edith Chen, Régine Debrosse, Paula J. Ham, Lauren C. Hoffer, Adam K. K. Leigh, Mesmin Destin

**Affiliations:** Northwestern University

**Keywords:** academic motivation, social support, inflammation, adolescents

## Abstract

Bolstering academic motivation is a high priority in school settings, but some evidence suggests this could take a toll on students’ physical health. To address this, this study compared the effects of an experimental manipulation of academic motivation alone (AM) to academic motivation enhanced with social support (SS+AM) on markers of inflammation in a sample of 80 high school 9^th^ graders. Outcomes included low-grade inflammation: C-reactive protein (CRP) and interleukin-6 (IL-6); a motivation measure; and grade point average (GPA), taken at baseline and follow-up (beginning and end of school year, respectively). Students in the SS+AM condition had lower levels of inflammation at follow-up (covarying baseline levels) compared to those in the AM condition. The two groups were equivalent on motivation and GPA at follow-up. This preliminary study suggests that incorporating social support into academic motivation programs has the potential to benefit inflammatory markers in young people while allowing them to maintain positive academic outcomes.

## Introduction

Keeping students focused on their educational futures has long been a high priority in American society, with the idea that education provides one route to improving one’s life circumstances. Consistent with this idea, increasing student motivation and persistence in academic settings are thought to represent key pathways to better educational outcomes for students (Duckworth, 2016; Oyserman, 2015).

Interventions focused on motivation and persistence are thought to increase student striving and self-control. In turn, these constructs have been linked to positive outcomes in life, such as higher incomes and better mental health (Moffitt et al., 2011; Brody, Yu, Miller, & Chen, 2016). At the same time, emerging evidence suggests that some students who expend high effort striving for academic success can experience a physical health cost of this success. Tradeoffs have been observed, whereby economic success and positive mental health in adulthood come at the expense of physical health for some individuals. For example, higher incomes, greater educational attainment, and lower levels of depression and substance use have been found in a subset of adults who at the same time were more likely to have diabetes, metabolic syndrome, higher allostatic load (a multi-system indicator of physiological risk), and faster epigenetic aging (DNA methylation profiles that reflects the discrepancy between a person’s biological and chronological age). These patterns are typically found in adults characterized as high strivers in adolescence (Brody et al., 2016; Brody et al., 2013; Miller, Yu, Chen, & Brody, 2015; Miller, Chen, Yu, & Brody, 2020), with effects being more pronounced among those from low-income backgrounds or individuals of color. Similarly, students of color with asthma who exhibit high self-control under stressful school conditions have less anxiety and depression, but also worse asthma inflammatory profiles compared to those with low self-control (Chen et al., 2019). This pattern has been termed “skin-deep resilience,” reflecting the idea that ‘above the skin,’ these young people appear to be doing well and achieving successes by many external metrics (e.g., earning good incomes, good mental health profiles). However, ‘below the skin,’ these same individuals appear to be struggling in terms of their physical health.

One hypothesis for why skin-deep resilience occurs is that the years of persistent high effort that students expend to achieve academic successes can take a physiological toll on them, particularly for students who grow up in environments with limited economic resources. In these environments, students often pursue academic goals while simultaneously confronting barriers such as financial challenges at home and underfunded schools (Duncan & Murnane, 2011; Chen, Brody, & Miller, in press). Furthermore, as students progress into higher education settings, their concentrated focus on academics and pursuit of success becomes increasingly solitary and associated with a weakening of important social ties, particularly for students of color and those from low-income neighborhoods (Destin, Rheinschmidt-Same, & Richeson, 2017). Often these students find themselves surrounded by fewer students from similar backgrounds, and are more likely to experience a lack of belonging, as well as discrimination and microaggressions (Johnson, Richeson, & Finkel, 2011; Cole & Omari, 2003). These experiences result in increasing feelings of social isolation. In addition, connections with their communities and families of origin might progressively weaken, as the life experiences of these students become increasingly removed from those with whom they grew up (Cole & Omari, 2003; Destin et al., 2017; Van Laar, Bleeker, Ellemers, & Meijer, 2014). Thus bolstering students’ academic motivation while also fostering social support might help prevent the emergence of skin-deep resilience. To test this question, we conducted a preliminary study to investigate whether the addition of an emphasis on social support (identifying important sources of social support and connection in students’ lives) to efforts to enhance academic motivation in a school setting would improve biomarkers of inflammation in a sample of high school students.

One commonly utilized approach to promoting academic motivation is through an exploration of students’ future identities. This approach is based on the notion that how people conceptualize their future is based on messages and experiences from their surrounding contexts, which shapes their current behaviors. For example, if students are surrounded by cues that lead them to picture a future identity involving a plausible and detailed route to college, they are more likely to work toward long-term academic goals (Destin & Oyserman, 2010; Oyserman, 2015). Identity-based motivation experiments have been found to improve academic motivation, engagement in schoolwork, and grades in comparison to students in control groups (Destin, 2017; Oyserman, Destin, & Novin, 2015; Oyserman, Bybee, & Terry, 2006). Other related efforts, such as getting students to consider how classroom topics are connected to their life goals and encouraging students to consider how school is connected with a sense of purpose also have increased academic achievement (Hulleman & Harackiewicz, 2009; Walton & Wilson, 2018; Yeager et al., 2014). However, connecting this literature to skin-deep resilience raises the question of whether these programs might be having unintended negative consequences for physiological processes or physical health. To our knowledge, the experimental effects of academic motivation programs on health-relevant biomarkers have not been previously tested.

In an initial effort to test our ideas, we conducted a preliminary study adding a social support component to a standard academic motivation program. Adding a social support emphasis is consistent with a large literature documenting that social support is beneficial to physical health (Cohen, 2004; Holt-Lunstad, Smith, & Layton, 2010; Uchino, Cacioppo, & Kiecolt-Glaser, 1996). This literature includes research linking social support specifically to markers of inflammation (see Uchino, 2006; Uchino et al., 2018 for reviews). In the present study, our key outcome variable was low-grade inflammation, given evidence of inflammation serving as one central biological pathway connecting childhood stress to the development of multiple chronic diseases in adulthood (Miller, Chen, & Parker, 2011); given the key role that inflammation plays in cardiovascular-related conditions such as obesity and atherosclerosis (Hotamisligil, 2006; Libby, Ridker, & Hansson, 2009); and given that markers of inflammation such as C-reactive protein (CRP) and interleukin 6 (IL-6) have been prospectively associated with risk for Type 2 diabetes, coronary heart disease, and myocardial infarctions and strokes (Ridker, 2003; Ridker, Rifai, Cook, Bradwin, & Buring, 2005; Ridker, Hennekens, Buring, & Rifai, 2000; Ridker, Rifai, Stampfer, & Hennekens, 2000; Pradhan, Manson, Rifai, Buring, & Ridker, 2001; Pradhan et al., 2002). We hypothesized that an experimental condition that included the addition of an emphasis on social support to an academic motivation condition (SS+AM) would produce lower levels of low-grade inflammation compared to AM alone in high school students. By specifically comparing these two conditions in this proof-of-concept study, we conducted a conservative test of the benefits of adding a social support component to an active condition that provides academic motivation in a school setting. Given that both conditions contained the AM component, we hypothesized that the two conditions would result in equivalent academic and motivation outcomes.

## Method

### Participants

Participants were 9^th^ graders (ages 14–15) recruited from one large diverse public high school. Ninth grade is a critical period during the transition to a new high school context. During this period, students are especially attentive to social and academic norms making this a time when experimental programs are more likely to exert a significant effect on how students conceptualize themselves within the school context and in relation to their goals (Yeager & Walton, 2011). The study was advertised at the school through flyers, announcements, and information sessions. Eighty students participated in the study (being a preliminary proof-of-concept study, this number reflected the students we were able to enroll across two waves of data collection, given that the program was optional and offered during after school or weekend hours). In order to welcome as many students as possible, and given the sample of young people who in general are healthy, we kept eligibility criteria to a minimum and did not have health or other exclusion criteria (other than restricting the study to 9^th^ graders). We also did not restrict the demographic composition of the sample in this initial study in order to allow for the possibility that SS+AM approaches might be beneficial to a wide range of students. Approval for this project was received from the university and school district Institutional Review Boards. Parents provided informed consent, students provided assent, and participating students were compensated $50 for study participation across the school year.

### Procedures

Participants attended a baseline assessment during the fall semester (September-November) to complete informed consent, questionnaires, and to receive a blood draw. All assessments occurred in the mid- to late-afternoon (after school hours). Participants were randomly assigned to one of two conditions, AM or SS+AM. Because both conditions were structured in the same way and contained overlapping content, participants were generally unaware of the condition to which they had been assigned. AM/SS+AM sessions were conducted in small groups shortly after the baseline assessment (sessions also occurred between September-November). In total, there were 4 sessions, each one-hour long. All were conducted within the space of one week during after school or weekend hours, and were led by graduate students or trained undergraduate research assistants. At the end of spring semester (April-May), participants completed the same set of measures in a follow-up assessment as they did at baseline.

### Experimental Conditions

AM: In keeping with many social psychological approaches to enacting change in educational settings (Yeager & Walton, 2011), experimental conditions were kept relatively brief and targeted students’ thoughts and beliefs. Ninth graders completed sessions that were facilitated in small groups at the high school outside of school time. The sessions were based on a previously validated future identity school-based curriculum (Oyserman, 2015). All sessions included a combination of engaging discussions and activities. The first session focused on introductions, expectations, and team building. In session two, students explored possible images of adulthood and related goals. In session three, students created and shared timelines drawing detailed connections and pathways from their current and proximal behaviors to their future identities. In the final session, students discussed facing everyday challenges and problems and reviewed specific information about high school courses and college requirements.

SS+AM: Students completed sessions with the same curriculum as students in the AM groups, however each discussion and activity included a specific emphasis on the role of important sources of social support and connection. For example, their introductions included a discussion of significant people in their lives, the activities related to goals and courses included detailed consideration of how to reach out for help, and the discussion of future pathways incorporated writing about how important people will remain a part of their lives.

### Measures

#### Low-grade inflammation.

Antecubital blood was collected into an 8.5mL serum separator tube. We measured two of the most commonly assessed biomarkers of inflammation, CRP and IL-6 (Del Giudice & Gangestad, 2018), both of which are associated with future type 2 diabetes, myocardial infarctions, and other cardiovascular events (Pradhan et al., 2001; Ridker et al., 2000; Ridker, 2003). CRP was measured in duplicate by high-sensitivity immunoturbidimetric assay on a Roche/Hitachi cobas c502 analyzer. IL-6, a cytokine that orchestrates inflammation, was measured in duplicate by electrochemiluminescence on a SECTOR Imager 2400A (MesoScale Discover). Raw values of each marker were log transformed to correct for their non-normal distribution. An inflammation composite was computed by standardizing and averaging CRP and IL-6 values, given the significant correlation between the two measures at baseline (r=.51, p<.001), and their interconnection biologically (IL-6 stimulates the production of CRP in the liver; Del Giudice & Gangestad, 2018). This approach is consistent with previous studies (Bertoni et al., 2010; Koukkunen et al., 2001).

#### Grade Point Average (GPA).

Cumulative unweighted GPA was provided at the end of the school year by the high school.

#### Motivation.

The Grit scale was used, which contains eight items probing the tendency to engage in sustained effort toward long-term goals and to maintain consistent interests and goals over time, rated on a 5 point scale (Duckworth & Quinn, 2009). This is a widely used, validated indicator of student perseverance toward goals that has been validated in this age group and is predictive of student achievement (Duckworth & Quinn, 2009; Duckworth, 2016). Studies have shown that short-term changes in response to experimental manipulation can be detected in this measure (Jones, Destin, & McAdams, 2018). Cronbach’s alpha ranged from .73–.75 across the two time points, and the correlation across time for this measure was .77. Higher scores indicate greater motivation.

#### Social support.

As a manipulation check, the Harter Social Support Scale for Children was administered, a widely used and extensively validated self-report instrument (Harter, 1985). Participants report the degree to which they perceive social support from others on a 4-point scale. Cronbach’s alpha ranged from .79–.83 across the two time points, and the correlation across time for this measure was .38 in this sample. Higher scores indicate greater perceived support.

#### Body mass index.

Height and weight were measured during the baseline session using a medical-grade balance beam scale with stadiometer. Body mass index (BMI) was calculated as weight in kilograms divided by height in meters squared.

### Statistical Analyses

T-tests were used to compare groups at baseline. Primary analyses were analyses of covariance (ANCOVA), which were used to test group differences at follow-up covarying baseline levels of outcome variables (this is statistically equivalent to testing change scores covarying baseline). In secondary analyses, BMI was also included as a covariate for inflammation outcomes. For GPA, a t-test was used because there was no baseline GPA available (given that GPAs came from high school records, and that the sample consisted of 9^th^ graders just starting high school at the time of the baseline assessment).

There was some incomplete participation and missing data in this sample. 86% of students attended all experimental sessions. At follow-up, seven students had moved schools, and we were unable to reach eight students. These 15 students did not have psychosocial or inflammation follow-up data, and hence were not included in analyses of these outcomes. However all students who had not moved schools were included in GPA analyses, given that these data came from school records.

## RESULTS

### Descriptive and Baseline Information

The sample was 41% male students, 46% White, 24% Black, 22% Latinx, and 5% Asian (the rest were ‘other’). Thirty-one percent were from lower socioeconomic status families (roughly defined in this study as the family living in an apartment rather than a house). The racial/ethnic and socioeconomic breakdown of the sample were comparable to the high school’s demographics (46% White, 27% Black, 18% Latinx, 6% Asian, 36% low-income). There were no differences between the two groups at baseline on demographic, inflammation, or psychosocial measures (all p’s >.05). Because the sample consisted of 9^th^ graders, we did not have GPA data from the high school at baseline. However, students did self-report their previous grades at baseline, and there were no differences at baseline between the two groups on self-reported grades (p>.3). In addition, there was no evidence that our sample differed from the overall student body of the high school in terms of GPA in their first semester of high school (p>.3)

#### Manipulation check.

To assess whether our experimental condition altered social support, we conducted a manipulation check using a paired samples t-test contrasting support scores from baseline to follow up in the SS+AM group. The SS+AM group was found to marginally increase in social support across the school year (baseline M=3.22, SD=.78, follow-up M=3.45, SD=.66, t=1.92, p=.06). In contrast, the AM only group did not show changes in social support across the year (t=1.21, p=.24).

### Primary Analyses

#### Low-grade inflammation.

**Figure 1:**
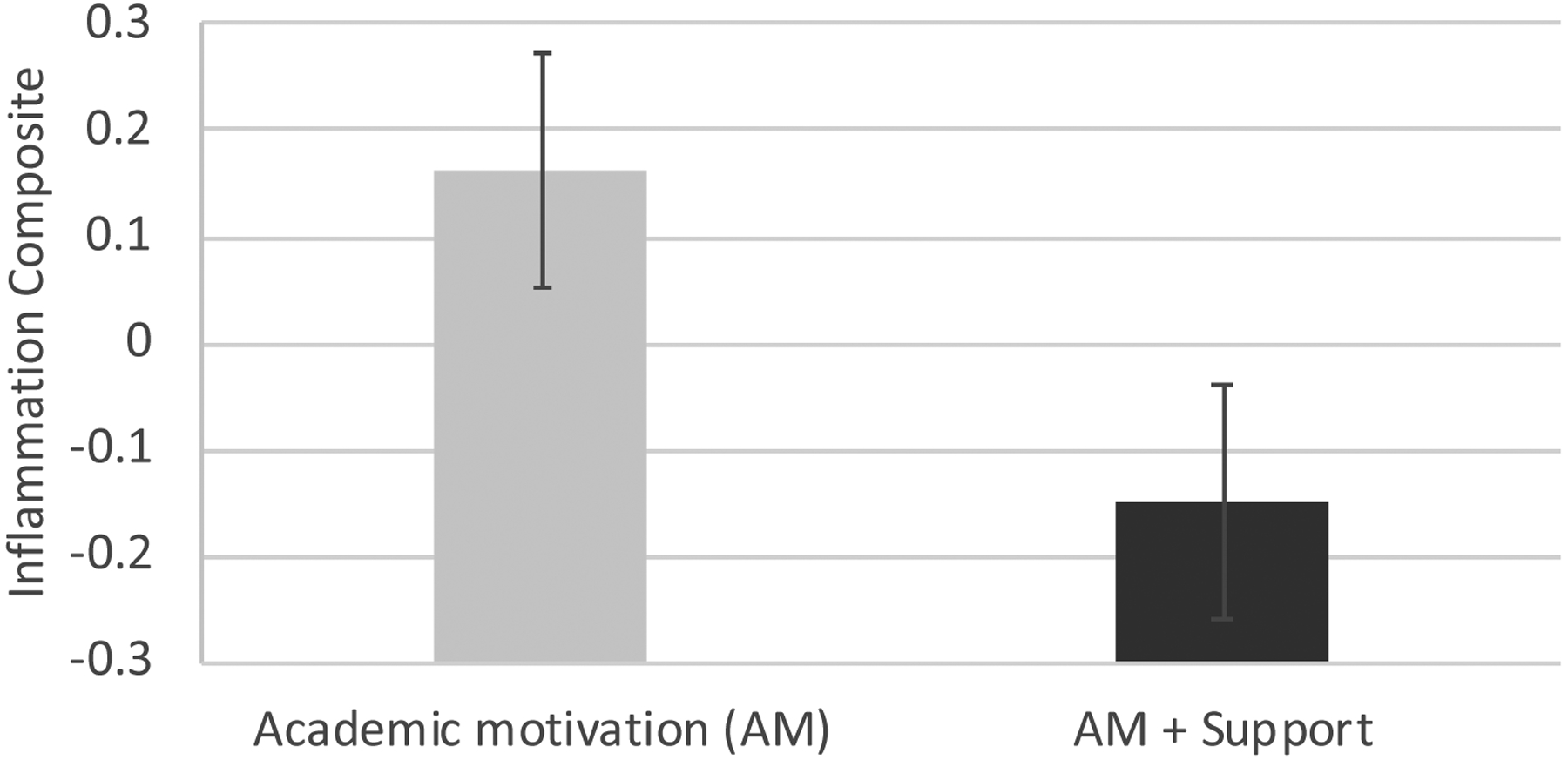
Inflammation composite (log-transformed, standardized, and averaged values of CRP and IL-6) at the end of the school year, adjusted for baseline levels, by experimental group. Error bars represent standard errors of the mean.

A significant effect of condition emerged for the inflammation composite (F=4.23, p=.044; AM estimated marginal mean at end of school year covarying baseline levels=.16, standard error of the mean, SEM=0.11. SS+AM mean= −.15, SEM=.10, Cohen’s d=.51). The SS+AM group had significantly lower levels of inflammation at follow-up, after covarying baseline levels, compared to the AM group (see Figure 1 and Table 1).

In secondary analyses, we controlled for BMI. The effect of condition remained significant after including this additional covariate, with the SS+AM group having significantly lower levels of inflammation at follow-up, after covarying baseline levels, compared to the AM group (F=4.42, p=.04; AM estimated marginal mean at end of school year covarying baseline levels =.16, SEM=.10. SS+AM mean= −.15, SEM=.10). When inflammatory markers were analyzed separately, this effect controlling for BMI was significant for CRP (F=4.98, p=.029; AM log-transformed mean=−.27, SEM=.06. SS+AM mean= −.44, SEM=.05), but not IL-6 (F=.29, p=.59; AM log-transformed mean=−.13, SEM=.04. SS+AM mean=−.16, SEM=.04).

#### Academic/psychological outcomes.

The AM and SS+AM groups were equivalent on end-of-year GPA (t=.361, p=.719; AM mean=3.15, SEM=.13. SS+AM mean=3.22, SEM=.13), and on follow-up motivation, covarying baseline levels (F=.044, p=.835; AM estimated marginal mean at end of school year covarying baseline levels =3.25, SEM=0.07. SS+AM mean=3.27, SEM=.07). See Table 1 for means and standard deviations of outcome variables by group.

## DISCUSSION

The results of this proof-of-concept experimental study suggest that adding an emphasis on social support to academic motivation efforts in a school setting has the potential to reduce low-grade inflammation without coming at a cost to academic outcomes in high school 9^th^ graders. Students who received a previously validated curriculum to improve academic motivation with an added emphasis on social support showed lower levels of low-grade inflammation at the end of the school year compared to AM alone, whereas the two groups did not differ on end-of-year motivation or grades. This design represented a conservative test of study hypotheses, as we compared two active conditions that were identical in terms of content related to academic motivation, but where one was supplemented with discussions about social support. To the best of our knowledge, this is the first experimental study of academic motivation that includes biomarkers of inflammation as an outcome.

We speculate that one reason for the inflammation differences may be that for some students, sustained academic motivation and academic successes are accompanied by feelings of pressure and sometimes have to involve largely independent efforts on the student’s part. This might occur if, for example, students’ families do not have much available time or experience to guide their high school student academically, or if their school lacks resources to adequately support the student in their academic endeavors. As a result, for some students, as they progress into higher education settings, their focus on academics and pursuit of success becomes more solitary and may be associated with a weakening of social ties (Destin et al., 2017). This may result in increasing feelings of social isolation for certain students who are high academic strivers. This persistent academic striving combined with perceived social isolation may be experienced by students as stressful, with resulting implications for stress-responsive physiological systems. Thus efforts to bolster social support in the context of school settings may help to strengthen students’ support networks and social connections as they face academic challenges, and potentially improve health-relevant physiological indicators, such as biomarkers of inflammation.

Our decision to evaluate outcomes toward the end of the academic year is in alignment with theoretical perspectives from social and educational psychology which suggest that educational contexts shape student experiences and outcomes *over time*. The study manipulation occurred during a specific developmental period (the transition to high school) when meaningful shifts in how young people interpret, experience, and engage with their learning contexts can get reinforced in ways that can sustain and amplify effects over time (Yeager & Walton, 2011). Our findings are consistent with previous research in which brief interventions in an educational context have been found to have lasting effects on outcomes, even years later (Oyserman et al., 2006; Yeager & Walton, 2011).

We found no differences at follow-up in motivation between the two groups, which was consistent with our predictions; however, it also appeared that there was little change in motivation over time. Other research on motivation during adolescence demonstrates a normative decline in levels of academic motivation and achievement across the adolescent years (Oyserman et al., 2006). The present study’s findings are consistent with the notion that often effective interventions are those that reduce or eliminate the drop in motivation and achievement, rather than expecting interventions to increase motivation (e.g., Oyserman et al., 2006).

In the present study, we focused on biomarkers of inflammation as an outcome given that our sample was young and non-clinical. Measuring inflammation in young people allows us to tap a key precursor to potential later adult diseases, given robust evidence that inflammation serves as an important pathway between early life psychosocial conditions and the development of multiple chronic diseases in adulthood (Miller et al., 2011). Biomarkers of inflammation measured in childhood or adolescence have health-relevance in being associated with clinical outcomes such as adiposity, blood pressure, metabolic syndrome, carotid intima-media thickness, lesions in the coronary artery, and premature coronary heart disease (Balagopal et al., 2011; Celermajer & Ayer, 2006; Groner, Joshi, & Bauer, 2006). In this study, we focused on two of the most commonly assessed biomarkers of inflammation in the literature, CRP and IL-6 (Del Giudice & Gangestad, 2018); when analyzed separately, effects were found to be more evident for CRP.

Strengths of this study include a theoretically designed set of materials for the student sessions, the conduct of this study in a real-world setting (a diverse public high school), and the biological markers of inflammation measured (rather than relying on self-reports of health). Limitations of this study include the small sample size. This sample was small because it was a preliminary proof-of-concept study. Future larger and more rigorous trials are needed to determine the replicability and robustness of these findings. This would include studies with larger samples sizes, and studies with more stringent exclusion criteria (e.g., medical and psychiatric conditions). Another limitation is the lack of a comparison group that received neither academic motivation nor social support. In part, this was done to align ourselves with school efforts to make academic support programs available to all students; one strength is that this then provides a more conservative study design, given that all students received an active condition. In addition, in this study, we opened participation to 9^th^ graders more generally, rather than limiting participation to only certain groups of students, such as those from low-income backgrounds or students of color. This was done because we thought the addition of social support might be helpful to a wide range of students. Consistent with this notion, there are some recent studies on skin-deep resilience that have documented effects across multiple racial/ethnic groups (Chen et al., 2020; Miller et al., 2020). Nonetheless, the majority of skin-deep resilience research suggests that the benefits seen in this study might be even more pronounced amongst students who come from low-income backgrounds or students of color. This needs to be empirically tested in future studies. Finally future studies should test effects on clinical health outcomes, on other academic outcomes, and examine whether longer periods of participation in the experimental conditions could produce more sustained effects.

This study was intended as a proof-of-concept of the idea that academic motivation efforts supplemented with a social support emphasis could potentially benefit biomarkers of inflammation in high school students. Future studies will need to test the reproducibility of these effects in larger, more rigorous trials. If replicated, such effects might suggest that we should be considering students more holistically, and broadening the content of academic motivation programs to be mindful of physical health while encouraging academic success in students.

## Figures and Tables

**Table 1.** Means and Standard Deviations of Outcome Variables at Baseline and Follow-Up by Group

Academic Motivation					Academic Motivation + Social Support			
	Baseline		Follow-Up		Baseline		Follow-Up	
	M	SD	M	SD	M	SD	M	SD
CRP (mg/L)	0.80	.96	0.77	0.76	0.72	1.21	0.51	0.62
IL-6 (pg/mL)	0.84	.69	0.88	0.52	0.92	0.89	0.88	0.66
GPA			3.15	0.76			3.22	0.79
Motivation	3.21	.55	3.24	0.60	3.14	0.56	3.27	0.55

*Note*: CRP=C reactive protein. IL-6=interleukin 6. GPA = cumulative grade point average at the end of the school year. Motivation = score on Grit questionnaire.
